# Correlation between *JAK2*, *STAT3*, and *CTLA4* Relative Gene Expressions and Oral Squamous Cell Carcinoma

**DOI:** 10.30476/dentjods.2024.100237.2205

**Published:** 2025-03-01

**Authors:** Hamideh Kadeh, Tayebeh Baranzehi, Milad Mollaali, Neda Maserat, Mohammad Javad Shahraki, Dor Mohammad Kordi-Tamandani

**Affiliations:** 1 Dept. Oral and Maxillofacial Pathology, Oral and Dental Disease Research Center, Faculty of Dentistry, Zahedan University of Medical Sciences, Zahedan, Iran; 2 Dept. of Biology, Faculty of Science, University of Sistan and Baluchestan, Zahedan, Iran

**Keywords:** Squamous Cell Carcinoma of Head and Neck, Janus Kinase 2, STAT3 Transcription Factor, CTLA-4 Antigen, Gene Expression

## Abstract

**Statement of the Problem:**

Oral squamous cell carcinoma (OSCC) is the eighth leading cause of cancer-related death worldwide. *JAK2* and *STAT3* primarily influence intrinsic tumor cell behavior, and *CTLA4* impacts the interplay between the tumor and the host immune system in the context of cancers. There is scarce information regarding the involvement and roles of *JAK2*, *STAT3*, and *CTLA4* genes in OSCC; however, the molecular mechanisms are still unclear.

**Purpose:**

This study examined the relationship between *JAK2*, *STAT3*, and *CTLA4* gene expression levels and OSCC in a group of patients in the southeast of Iran.

**Materials and Method:**

This cross-sectional study was conducted in which the relative gene expression levels of *JAK2*, *STAT3*, and *CTLA4* were compared between 23 oral paraffin tissue blocks collected from OSCC patients and 20 fresh gingival tissues collected from healthy individuals. The Real-Time quantitative PCR (RT-qPCR) assay was employed to assess relative gene expression levels. SPSS 27 was employed to perform statistical analyses.

**Results:**

Significant differences were found between OSCC patients and healthy individuals concerning gene expression levels of *JAK2* (2.4-fold, *p*< 0.0001), *STAT3* (2.32-fold, *p*< 0.0001), and *CTLA4* (4.09-fold, *p*< 0.0001). Additionally, there were significant positive correlations among *JAK2-STAT3* (ρ= 0.667, *p*< 0.001), *JAK2-CTLA4* (ρ= 0.771, *p*< 0.001), and *STAT3-CTLA4* (ρ= 0.635, *p*= 0.001) co-expressions. Moreover, gender, age groups, and tumor locations did not significantly correlate with the expression levels of these genes (*p*> 0.05). Nevertheless, significant differences occurred between histopathological grades and the gene expression levels of *JAK2* (*p*< 0.001), *STAT3* (*p*= 0.001), and *CTLA4* (*p*< 0.001)

**Conclusion:**

The overexpression of *JAK2*, *STAT3*, and *CTLA4* can be considered triggers for OSCC development. It may be beneficial to conduct future research on OSCC by considering downstream genes involved in the *JAK2*/*STAT3*/*CTLA4* axis.

## Introduction

Oral squamous cell carcinoma (OSCC) is the overwhelmingly prevalent type of oral cancer; its 5-year survival rate is about 50-60% [ 1 ]. OSCC is typically asymptomatic in the earliest stages, and most patients are diagnosed after it becomes more advanced [ 2 ]. Many risk factors, such as low antioxidant diet, human papillomavirus infection, UV exposure, chronic local trauma, potentially malignant lesions of the mouth, and suppression of the immune system are associated with oral cancer [ 3 ]. 

The genes in the signal transducer and activator of transcription (STAT) family provide necessary instructions for proteins involved in parts of the cell’s chemical signaling pathways. When chemical signals activate STAT proteins, they travel to the nucleus and bind to regulatory regions of genes, causing these genes to turn on or off [ 4 ]. The *STAT3* gene resides at the top of the STAT gene family; a phosphorylated *STAT3* protein forms a homodimer or heterodimer regarding cytokines, acting as a transcription activator [ 5 ]. In addition to mediating numerous gene expressions, *STAT3* regulates the cell’s response to external stimuli, so it has been observed in multiple cellular mechanisms (such as apoptosis and cell growth) [ 6 ]. Unphosphorylated STATs are in the cytoplasm; binding of cytokines to Janus kinase (JAK) receptors causes them to deform and dimerize, change their position, and become activated. Activated JAKs induce phosphorylation and activation of tyrosine kinases and provide the basis for the activity of STAT cytoplasmic transcription factors. Phosphorylation of tyrosine in STAT by JAKs causes the SH2 domain to bind to phosphorylated tyrosine. In response to tyrosine phosphorylation, *STAT3* forms a dimer and is activated; these dimers are transported into the nucleus and attached to TTCN2-4GAA-agreed GAS motifs (located at the target gene promoter) to activate transcription [ 7 ].

Cytotoxic T-lymphocyte-associated protein 4 (*CTLA 4*), also known as CD152, is an immunosuppressive receptor in T cells [ 8 ]. *CTLA4* acts as a negative regulator of T cells involved in antitumor immune responses, and its blockade can enhance immune responses and repel tumors; it has been hypothesized that *CTLA4* may reduce antitumor responses and increase the risk of cancer by raising the T cell activation threshold in the early stages of tumorigenesis [ 9 ]. Complete loss of *CTLA4* in mice induces lethal autoimmunity during the first three weeks after birth, indicating the vital role of *CTLA4* in inhibiting autoimmune responses [ 10 ].In certain parts of Iran, such as Sistan and Baluchestan Province, OSCC is more prevalent than in other parts due to its proximity to Pakistan and India [ 11 ]. Evaluation of gene expression can provide insightful information about the tumor microenvironment. Until now, to our knowledge, the *JAK2*/*STAT3*/*CTLA4* axis has not been investigated for tissue-specific gene expression at the mRNA levels in OSCC. Hence, our study aimed to examine the association between *JAK2*, *STAT3*, and *CTLA4* relative gene expressions and OSCC in the southeast of Iran.

## Materials and Method

### Sample selection

In the present study, 23 paraffin blocks from OSCC patients and 20 fresh gingival biopsy samples were collected from the Faculty of Dentistry at Zahedan University of Medical Sciences in 2019 and 2020, following pathobiological evaluations. Informed consent was obtained from all participants. Also, the Zahedan University of Medical Sciences Ethics Committee ethically approved this study (Approval ID: IR.ZAUMS.REC. 1397.321). The clinical and demographic characteristics of the participants are presented in Table 1.

**Table 1:** Demographic and clinicopathological characteristics information of OSCC and control groups

	OSCC (n=23)		Controls (n = 20)	
Mean age±SD (year)	58.78±12.84		41.40 ± 12.15	
Age groups	Male (%)	Female (%)	Male (%)	Female (%)
50>	1 (4.35)	5 (21.73)	4 (20)	11 (55)
50≤	6 (26.09)	11 (47.83)	2 (10)	3 (15)
Histopathological grades				
Well-differentiated	3 (13.04)	9 (39.13)		
Moderately-differentiated	4 (17.39)	6 (26.09)		
Poorly-differentiated	-	1 (4.35)		
Tumor location				
Mandibular gingiva	4 (17.39)	6 (26.09)		
Tongue	2 (8.70)	4 (17.39)		
Buccal mucosa	-	4 (17.39)		
Palate	1 (4.34)	-		
Maxilla gingiva	-	2 (8.70)		

Abbreviation: SD, Standard deviation

### Preparation of tissue sections

A microtome instrument was employed to slice paraffin blocks containing the oral tissues into 10 μm sections. After that, they were deparaffinized using the xylene-ethanol method [ 12 ]. For fresh gingival tissues, a homogenizer was employed to homogenize them. 

### RNA extraction and cDNA synthesis

RNA was extracted from the deparaffinized OSCC tissues and fresh gingival tissues using Total RNA Extraction Kit^TM^ (Cat. No. A101231, Pars Tous Co., Mashhad, Iran), according to the manufacturer’s instruction. In addition, RNA purity (using Absorbance 260 nm / Absorbance 280nm) and RNA concentration (using Absorbance 260nm×Factor 40) were measured via ScanDrop^®^ 250 spectrophotometer (Analytik Jena Co., Jena, Germany). Then, the RNA was electrophoresed on 1% agarose gel to determine its integrity. Using 10 μg of RNA, the cDNA was synthesized via Easy™ cDNA Synthesis Kit (Cat. No. A101161, Pars Tous Co., Mashhad, Iran), according to the manufacturer’s instruction.

### Real-Time quantitative PCR (RT-qPCR) assay

RealQ Plus 2x Master Mix Green High Rox^TM^ (Cat. No. A325402, Ampliqon Co., Odense, Denmark) was used for the SYBR Green-based Real-Time quantitative PCR (RT-qPCR) assay. StepOne^TM^ Real-Time PCR System (Applied Biosystems Co., San Francisco, CA, USA) instrument was applied to estimate the involved cDNA using RT-qPCR. The sequences of the primers which applied for the RT-qPCR assay was included: *JAK2* forward: 5ʹ-CCCTCCATTTCTGTCATC-3ʹ; *JAK2* reverse: 5ʹ-AAGCAGGCAACAGGAACAAG-3ʹ; *STAT3* forward: 5ʹ-GACTCTCAATCCAAGGGGC-3ʹ; *STAT3* reverse: 5ʹ-CCTCTGCCGGAGAAACAG-3ʹ; *CTLA4* forward: 5ʹ-CACAAGGCTCAGCTGAACCT-3ʹ; *CTLA 4* reverse: 5ʹ-AGGTGCCCGTGCAGATGGAA-3ʹ; *RNA 18S* forward: 5ʹ-GTAACCCGTTGAACCCCA-TT-3ʹ; and *RNA 18S* reverse: 5ʹ-CCATCCAATCGGT-AGTAGCG-3ʹ. *RNA 18S* was chosen as the housekeeping gene. The following thermal cycling parameters were used for each RT-qPCR reaction: initial denaturation at 95°C for 10 min; 40 cycles of 95°C for 15 s, annealing (*JAK2*: 59°C, *STAT3*: 58°C, *CTLA4*: 62°C, and *RNA 18S*: 60°C) for 1 min; also, the melting curve was obtained through 58-95°C. The 2^-ΔΔCT^ method [ 13 ] was considered to calculate relative gene expression. 

### Statistical analysis

**Figure 1:**
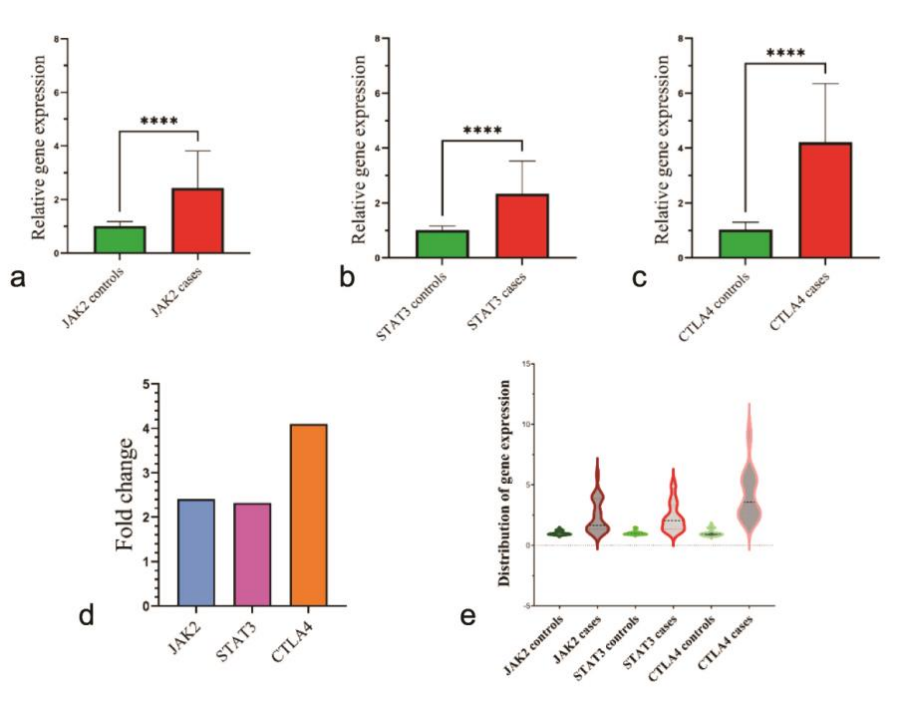
Gene expression status. The a, b, and c panels depict the relative expression of genes between cases and controls (mean expression ± SD) for *JAK2*, *STAT3*, and *CTLA4*, respectively. In the case group, gene expression levels are higher than in the healthy group for all genes. Panel d shows fold change for *JAK2* (2.4-fold), *STAT3* (2.32-fold), and *CTLA4* (4.09-fold). The violin plot shows the overall distribution of gene expression for each gene (panel e). Asterisks (****) means statistical significance at *p* < 0.000

Microsoft Excel 2021 was used to calculate the gene expression levels for each participant. Data were evaluated through SPSS 27 software to compute Shapiro-Wilk, Mann-Whitney U, Spearman’s Correlation Coefficient, and Kruskal Wallis tests. In addition, Figure 1 has been designed through GraphPad Prism 9.5.1 software. Statistical significance was established at *p*< 0.05 for all tests.

## Results

### Primary evaluation

RNA extraction and cDNA synthesis were performed from 20 tissues of healthy individuals and 23 tissues of OSCC patients, and amplifying the cDNA with *JAK2*, *STAT3*, and *CTLA4* sequence-specific primers obtained PCR products with the desired amplicon in 2% agarose gel electrophoresis. Patients’ tissues and tissues from healthy individuals expressed all these genes. In addition, RT-qPCR was used to determine whether gene expression differed between OSCC patients and healthy individuals, data were normalized to the internal control gene, RNA 18S.

### Normal distribution status of genes

Since there are fewer than 50 samples in this study, the Shapiro-Wilk test is more robust than other normality tests [ 14 ]; therefore, this test was carried out to determine the normal distribution of gene expression values. The results of the test revealed that none of these three genes followed a normal distribution (*p* < 0.001). Consequently, non-parametric tests will be used for subsequent statistical tests.

### Gene expression findings

There was a statistically significant association for relative gene expression in comparing tissue samples between OSCC patients and healthy groups for the *STAT3*, *JAK2*, and *CTLA4* genes (Table 2). Compared to normal subjects, OSCC patients expressed significantly more *CTLA4* (*p*< 0.0001). Additionally, OSCC patients’ tissues had significantly higher mRNA levels of *JAK2* (*p* < 0.0001) and *STAT3* (*p* < 0.0001) compared with normal tissues. In Supplementary Table 1, the mean of gene expression was provided for clinicodemographic features of healthy individuals and patients. This showed that females have slightly higher expressed genes in the control group. In the case group, the males had a higher expression for *JAK2* and *CTLA4*, although *STAT3* had a higher expression in females. In the control group, individuals 50≤ years old had higher expression of genes than those who were 50> years old; but in the case group, the pattern was reversed and only *STAT3* had high expression in 50≤ years old. Regarding histopathological grades in the case group, in all genes, the poor condition had the highest expression levels. Finally, the most affected site in patients was maxilla gingiva, which has the highest expression level for all genes.

**Table 2:** Comparison of *JAK2*, *STAT3*, and *CTLA4* relative gene expressions in case and control groups using the Mann–Whitney U test

Genes	Status	N	Mean Rank	Sum of Rank	*U*-value	*Z*-value	*p* Value
*JAK2*	Case	23	30.98	712.50	23.500	-5.029	< 0.0001****
	Control	20	11.68	233.50			
*STAT3*	Case	23	31.39	722.00	14.000	-5.260	< 0.0001****
	Control	20	11.20	224.00			
*CTLA4*	Case	23	32.00	736.00	0.000	-5.601	< 0.0001****
	Control	20	10.50	210.00			

**** Significant at *p* < 0.0001 level

**Supplementary Table 1:** The mean of gene expressions in OSCC and control groups in clinicodemographic features

		OSCC (n=23)			Control (n=20)		
		*JAK2* Expression (mean)	*STAT3* Expression (mean)	*CTLA4* Expression (mean)	*JAK2* Expression (mean)	*STAT3* Expression (mean)	*CTLA4* Expression (mean)
Gender	Male	2.731	2.297	5.017	0.936	0.993	0.965
	Female	2.297	2.361	3.863	1.044	1.016	1.056
Age groups	50>	1.776	2.136	3.556	1.030	1.023	1.086
	50≤	2.660	2.413	4.446	0.957	0.968	0.857
Histopathological grade	Well-differentiated	1.331	1.564	2.604			
	Moderately-differentiated	3.408	2.994	5.610			
	Poorly-differentiated	5.830	5.132	9.583			
Tumor location	Mandibular gingiva	2.361	2.172	4.218			
	Tongue	2.457	2.250	4.255			
	Buccal mucosa	2.285	2.662	3.421			
	Palate	1.498	1.580	3.118			
	Maxilla gingiva	3.441	3.199	6.206			

The expression levels of each gene between the two groups are illustrated in Figure 1 (a, b, and c panels); panel d shows the fold changes (mean expression of cases/ mean expression of controls) for each gene; and panel e shows the distribution of gene expression status. According to the results of the expression analysis, there was a statistically significant difference between cases and healthy controls for each gene; *JAK2* showed a 2.4-fold difference, *STAT3* displayed a 2.32-fold difference, and *CTLA4* showed a 4.09-fold difference. This means that the *CTLA4* gene had the highest expression in comparison to the *JAK2* and *STAT3* genes. In addition, the violin plot indicated that there was a notable difference in the distribution of gene expression within the case and the control groups; for all genes, the pattern of distribution in the case group was more non-uniform than the control group.

### Gene-gene and gene-clinicopathology relationships

As part of the correlation analysis of OSCC patients, the correlation among *JAK2-STAT3*, *JAK2-CTLA4*, and *ST-AT3-CTLA4* co-expression was investigated using Spearman’s Correlation Coefficient test (Table 3). According to Table 3, positive correlations could be found for *JAK2-STAT3* (0.667, *p*< 0.001), *JAK2-CTLA4* (0.771, *p*< 0.001), and *STAT3-CTLA4* (0.635, *p*= 0.001). Consequently, the highest gene-gene correlation was observed in *JAK2-CTLA4* in the patient group.

**Table 3:** Summarized results of Spearman’s Correlation Coefficient test for evaluating co-expression among OSCC patients (n= 23)

		*JAK2*	*STAT3*	*CTLA4*
*JAK2*	Spearman's rho	1.000	0.667	0.771
	*p* value	-	< 0.001***	< 0.001***
*STAT3*	Spearman's rho	0.667	1.000	0.635
	*p* value	< 0.001***	-	0.001**
*CTLA4*	Spearman's rho	0.771	0.635	1.000
	*p* value	< 0.001***	0.001**	-

** Significant at *p* < 0.01;

*** Significant at *p* <0.001

Furthermore, the association between relative gene expression and clinicopathological variables was evaluated in OSCC patients using the Kruskal-Wallis test (Table 4). The results indicated that only histopathological grades are associated with elevated expression of *JAK2*, *STAT3*, and *CTLA4* genes. Other characteristics including gender, age groups, and tumor localization are not linked to the upregulation of the genes.

**Table 4:** Summary of Kruskal-Wallis test results comparing gene expression and clinicodemographic characteristics among OSCC patients (n = 23)

	*JAK2* expression			*STAT3* expression			*CTLA4* expression		
	χ^2^	df	*p* value	χ^2^	df	*p* value	χ^2^	df	*p* value
Histopathological grade	17.106	2	< 0.001***	13.327	2	0.001**	16.624	2	< 0.001***
Tumor location	0.224	4	0.994	2.174	4	0.704	1.012	4	0.908
Gender	1.368	1	0.242	0.004	1	0.947	2.161	1	0.147
Age groups	1.865	1	0.172	0.177	1	0.647	0.490	1	0.484

Abbreviation: df, degrees of freedom. χ^2^, Chi-Square;

** Significant at *p* < 0.01;

*** Significant at *p* <0.001

## Discussion

This study assessed the expression levels of the *JAK2*, *STAT3*, and *CTLA4* genes in OSCC patients by utilizing RT-qPCR. A significant increase in the expression levels of *JAK2* (2.40-fold), *STAT3* (2.32-fold), and *CTLA4* (4.09-fold) has been observed in OSCC patients compared to healthy individuals, as shown in Table 2. RT-qPCR results of a cross-sectional study showed that *JAK2* was decreased and *STAT3* elevated gene expression level in breast cancer; there was also a positive correlation between *JAK2* and *STAT3* expression [ 15 ]. According to the Spearman test’s results (Table 3), there is a strong positive correlation between the expression levels of these three genes. Hence, if one of these genes increases/decreases, the other gene will decrease/ decrease correspondingly. Our findings are consistent with those of other studies. For example, in tumor-associated B cells, *STAT3* promotes *CTLA4* expression in a JAK-dependent mechanism [ 16 ], while in Treg cells, *STAT3* promotes *CTLA4* expression via *IL-10* [ 17 ]. Interestingly, there was no significant correlation between age groups, gender, or tumor location with the expression of *JAK2*, *STAT3*, and *CTLA4* in patients. However, in histopathological grades, a significant correlation was observed; poor status had the highest expression levels of these three genes. The mean expression of *STAT3* in women was more than in men, but the pattern was reversed for *JAK2* and *CTLA4*. For *CTLA4*, the gene expression ratio in men versus women was 1.30:1, and for *JAK2* it was 1.19:1, but for *CTLA4* the ratio was 1:0.97. *STAT3* indirectly induces the expression of immune checkpoint molecules by exerting influence on numerous signaling pathways through which it is expressed [ 18 ]. As a general rule, when *STAT3* is activated in cancer cells, it causes a change in the function of proteins that regulate and control the expression of inflammation genes by affecting the function of secretory proteins [ 19 ]. Possibly, this feature of *STAT3* explains our results. Elevated gene expression of *STAT3* has been observed in a broad spectrum of conditions, such as prostate cancer [ 20 ], lung cancer [ 21 ], and OSCC [ 22 ]. Activating the *STAT3*, associated with increased *STAT3* tyrosine phosphorylation, causes cell proliferation, and differentiation in OSCC [ 23 ]. However, *STAT3* inactivation is associated with immortality and metastatic potential in oral epithelial cells [ 24 ]. 

*CTLA4* has some interactions with the JAK/STAT pathway. Thomas *et al.* [ 25 ] examined various mechanisms and found that cancer cells use diverse approaches to promote the JAK/STAT pathway; for head and neck SCC, tumor cells express *CTLA4*, which phosphorylates the *STAT3* gene; thus, *CTLA4* can positively correlate with *STAT3*, which is similar to our results. Studies show that increased expression of *CTLA4* is associated with several cancers. For instance, a study by Erfani *et al.* [ 26 ] demonstrated a meaningful relationship between *CTLA4* gene expression and laryngeal SCC. However, another research by Erfani *et al.* [ 27 ] on *CTLA4* expression in non-small cell lung cancer did not reveal a significant correlation. Also, Adam *et al.* [ 28 ] stated that there was no relationship between patients’ age and sex and *CTLA4* expression in lung cancer. In our study, *CTLA4* expression levels were 25% higher in those aged 50 and older than in those younger than 50 years of age; furthermore, *CTLA4* expression was substantially increased in men compared to women, but the correlation was not significant. Our findings were in line with the Padma *et al.* study [ 29 ], which evaluated the effect of histopathological grades on OSCC severity. However, Moreira *et al.* [ 30 ] reported that *CTLA4* expression did not correlate with OSCC patients’ survival rate. In our study, although the survival rate was not assessed, *CTLA4* expression was significantly elevated in conditions with poor differentiation compared with conditions with well and moderate differentiation. 

The development of *JAK2*/*STAT3*-selective inhibitors for treating OSCC is currently underway [ 31 ]. The licochalcone C [ 32 ], licochalcone D [ 33 ], and licochalcone H [ 34 ] may promote apoptosis in OSCC cells by inhibiting *JAK2*, which inhibits the *JAK2*/*STAT3* signaling pathway and reduces cell growth. *CTLA4* monoclonal antibodies have been validated as therapeutic agents and are effective for treating lung and skin neoplasms [ 35 ] and recently in oral cancer [ 36 ]. In light of this, these inhibitors can be considered effective treatment options for oral cancer.

Our study had some limitations and challenges. The study was designed between OSCC patients and healthy individuals, the recommended approach is to use a paired sampling procedure (it means that both cancerous tissues and healthy tissues are obtained from the same individual); we will be able to observe expression changes more accurately under diverse circumstances using this procedure. Oral cancer development can be exacerbated by intervening factors such as smoking, alcohol, opioids, and oral hygiene status; these factors may partially affect gene expression levels in the OSCC microenvironment; we were unable to access this information regarding patients. Our study had a limited sample size; more extensive samples would be beneficial in future studies. Our study employed a SYBR Green-based RT-qPCR assay with site-specific primers, which is a routine method to assess gene expression; however, future studies should also consider the TaqMan-based RT-qPCR assay, which utilizes site-specific probes and is more sensitive. Additionally, other techniques such as Western blot should likewise be employed in future studies for the determination of the expression of the genes at their protein levels. Furthermore, future studies of OSCC are also expected to focus on analyzing gene expression across the various sections of the oral cavity.

## Conclusion

Our study indicated that *JAK2*, *STAT3*, and *CTLA4* expression is markedly upregulated in OSCC tissues in comparison with healthy tissues, highlighting that these genes might be involved in OSCC progress. However, elevated expression of these genes has not been proven to correlate with clinical parameters inside the patient group (except histopathological grade). There is potential for these genes to provide a new avenue for the development of personalized therapeutic agents to treat patients with OSCC. However, further investigations should be undertaken to determine how these genes might act as contributory factors to OSCC severity.Acknowledgments

The authors want to thank all participants for their participation in the study. This study received financial support from the Zahedan University of Medical Sciences with grant number 8695.
